# Supplementary material for: The Systems Biology Research Tool: evolvable open-source software
Source: BMC Syst Biol. 2008 Jun 29;2:55. doi: 10.1186/1752-0509-2-55 (PMC2446383; doi:10.1186/1752-0509-2-55)
Supplement: Additional file 1 — SBRT Archive. An archive of the current version of the Systems Biology Research Tool. [file 1752-0509-2-55-S1.zip › sbrt-1.4.0/doc/developers_guide/api/sbrt/kernel/fba/class-use/ChemRxn.html]

Uses of Interface sbrt.kernel.fba.ChemRxn


|  |  |  |
| --- | --- | --- |
| **Overview** \| **Package** \| **Class** \| **Use** \| **Tree** \| **Deprecated** \| **Index** \| **Help** | |  |
| PREV   NEXT | **FRAMES**    **NO FRAMES**     **All Classes** |  |


---


## **Uses of Interface sbrt.kernel.fba.ChemRxn**

| Packages that use ChemRxn | |
| --- | --- |
| **sbrt.kernel.fba** | Provides classes and interfaces for creating objects relevant to Flux Balance Analysis. |
| **sbrt.shell.mng.fba** | Provides classes and interfaces for creating Flux Balance Analysis process managers. |
| **sbrt.shell.text.fba** | Provides classes and interfaces for defining formats for objects relevant to Flux Balance Analysis. |

Uses of ChemRxn in sbrt.kernel.fba

| Classes in sbrt.kernel.fba that implement ChemRxn | |
| --- | --- |
| `class` | `AbstractRxn`             This class is a skeleton implementation of ChemicalRxn. |
| `class` | `ExchangeRxn`             This class is used to represent exchange reactions in Flux Balance Analysis. |
| `class` | `IrrevRxn`              This class is used to represent irreversible chemical reactions. |
| `class` | `ReversibleRxn`             This class is used to represent reversible chemical reactions. |
| `class` | `SinkRxn`             This class is used to represent sink reactions in Flux Balance Analysis. |
| `class` | `SourceRxn`             This class is used to represent chemical species sources in Flux Balance Analysis. |

| Methods in sbrt.kernel.fba that return ChemRxn | |
| --- | --- |
| `ChemRxn` | `Fluxome.getRxn(java.lang.String reactionName)`             Returns the chemical reaction in this fluxome with the provided name. |
| `ChemRxn` | `IrrevRxnFluxomeV1.getRxn(java.lang.String reactionName)`             Returns the chemical reaction in this fluxome with the provided name. |

| Methods in sbrt.kernel.fba that return types with arguments of type ChemRxn | |
| --- | --- |
| `java.util.Map<java.lang.String,? extends ChemRxn>` | `Fluxome.getInternalRxns()`             Returns the set of all internal chemical reactions contained in this fluxome. |
| `java.util.Map<java.lang.String,ChemRxn>` | `IrrevRxnFluxome.getReverseRxns(java.lang.String reactionName)`             Returns the set reactions in this fluxome that are stoichiometrically equivalent to the provided reaction in reverse. |
| `java.util.Map<java.lang.String,ChemRxn>` | `IrrevRxnFluxomeV1.getReverseRxns(java.lang.String reactionName)`             Returns all of the reactions in this fluxome that are stoichiometrically equivalent to the provided reaction in reverse. |
| `java.util.Map<java.lang.String,ChemRxn>` | `Fluxome.getRxns()`             Returns the set of all chemical reactions contained in this fluxome. |
| `java.util.Map<java.lang.String,ChemRxn>` | `IrrevRxnFluxomeV1.getRxns()`             Returns all chemical reactions contained in this fluxome. |
| `java.util.Map<java.lang.String,ChemRxn>` | `Fluxome.getRxns(java.util.Collection<java.lang.String> rxnNames)`             Returns the chemical reactions with the provided names. |
| `java.util.Map<java.lang.String,ChemRxn>` | `IrrevRxnFluxomeV1.getRxns(java.util.Collection<java.lang.String> rxnNames)`             Returns the chemical reactions with the provided names. |
| `java.util.Map<java.lang.String,? extends ChemRxn>` | `CatalyzedFluxome.getRxns(java.lang.String catalyst)`             Returns the chemical reactions contained in this fluxome that are catalyzed by the provided catalyst. |

| Methods in sbrt.kernel.fba with parameters of type ChemRxn | |
| --- | --- |
| `boolean` | `IrrevRxn.isEquivalent(ChemRxn rxn)`              Indicates if the provided chemical reaction is stoichiometrically equivalent to this reaction. |
| `boolean` | `ReversibleRxn.isEquivalent(ChemRxn rxn)`              Indicates if the provided chemical reaction is stoichiometrically equivalent to this reaction. |
| `boolean` | `SourceRxn.isEquivalent(ChemRxn rxn)`             Indicates if the provided chemical reaction is stiochiometrically equivalent to this source reaction. |
| `boolean` | `SinkRxn.isEquivalent(ChemRxn rxn)`             Indicates if the provided chemical reaction is stoichiometrically equivalent to this sink reaction. |
| `boolean` | `ChemRxn.isEquivalent(ChemRxn rxn)`             Indicates if this chemical reaction is stoichiometrically equivalent to the provided reaction. |
| `boolean` | `IrrevRxn.isReverse(ChemRxn rxn)`              Indicates if the provided chemical reaction is the reverse of this reaction. |
| `boolean` | `ReversibleRxn.isReverse(ChemRxn rxn)`             Returns `false`, since no reaction can be the "reverse" of a reversible reaction. |
| `boolean` | `SourceRxn.isReverse(ChemRxn rxn)`             Indicates if the provided chemical reaction is the reverse of this source reaction. |
| `boolean` | `SinkRxn.isReverse(ChemRxn rxn)`             Indicates if the provided chemical reaction is the reverse of this sink reaction. |
| `boolean` | `ChemRxn.isReverse(ChemRxn rxn)`             Indicates if the provided chemical reaction is the reverse of this reaction. |

Constructor parameters in sbrt.kernel.fba with type arguments of type ChemRxn

`IrrevRxnFluxomeV1(java.util.Map<java.lang.String,ChemRxn> rxns)`
  
          Constructs a new irreversible reaction fluxome from the
provided internal and exchange reactions.

`IrrevRxnFluxomeV1(java.util.Map<java.lang.String,ChemRxn> rxns,
java.util.Map<java.lang.String,Or<And<java.lang.String>>> catalysts)`
  
          Constructs a new irreversible reaction fluxome from the
provided internal and exchange reactions and catalysts.

Uses of ChemRxn in sbrt.shell.mng.fba

| Methods in sbrt.shell.mng.fba that return types with arguments of type ChemRxn | |
| --- | --- |
| `static RomoFile<java.lang.String,ChemRxn>` | `WwFluxomeReducerManager.getOutputFile(java.lang.String fileName)`             Returns a file to which the chemical reactions of a fluxome can be written. |
| `static RomoFile<java.lang.String,ChemRxn>` | `WwFluxomeReducerManager.getOutputFile(java.lang.String fileName, java.io.PrintWriter out)`             Returns a file to which the chemical reactions of a fluxome can be written. |

Uses of ChemRxn in sbrt.shell.text.fba

| Methods in sbrt.shell.text.fba that return ChemRxn | |
| --- | --- |
| `ChemRxn` | `IrrevRxnFormatV1.parse(java.lang.String reaction)`             Parses the provided string and returns its corresponding chemical reaction. |

| Methods in sbrt.shell.text.fba that return types with arguments of type ChemRxn | |
| --- | --- |
| `PipeListFormat<ChemRxn>` | `IrfFormatV1.getListFormat()`             Returns the format used for lists of reactions. |

| Methods in sbrt.shell.text.fba with parameters of type ChemRxn | |
| --- | --- |
| `java.lang.String` | `IrrevRxnFormatV1.format(ChemRxn rxn)`             Returns a formatted string representation of the provided irreversible chemical reaction. |

---


|  |  |  |
| --- | --- | --- |
| **Overview** \| **Package** \| **Class** \| **Use** \| **Tree** \| **Deprecated** \| **Index** \| **Help** | |  |
| PREV   NEXT | **FRAMES**    **NO FRAMES**     **All Classes** |  |


---
